# Society of Sanitary and Moral Prophylaxis

**Published:** 1910-05

**Authors:** Prince A. Morrow

**Affiliations:** 66 West 40th Street


### Society of Sanitary and Moral Prophylaxis.

### ORIGIN OF THIS MOVEMENT—ITS OBJECTS, AIMS AND METHODS OF WORK.

*The Origin.*—An international congress for the study and prevention of the diseases growing out of the social evil, in which every civilized country in the world was represented, was held in Brussels in 1902. The deliberations of this congress crystallized into the conviction that the preventive measures hitherto employed were insufficient, and that the whole question should be studied anew from a broader standpoint, and with special reference to the social conditions involved in the causation of these diseases. Especial recognition was given to the fact that moral as well as medical issues were involved in the problem of prevention.

This congress recommended that societies of sanitary and moral prophylaxis should be organized in all countries for the study of the best means of every order, moral, social, legislative, as well as medical, to be employed in the prophylaxis of these diseases. Such societies have been organized in Germany, France, Holland and other European countries. In Germany the society numbers nearly 5000 members, with twenty or more branches; in France, about 1000 members. These societies include in their membership ministers of state and other high public functionaries, representatives of the bar and church and men and women engaged in social work, besides practically all leading members of the medical profession.

The American society was organized in this country in New York City, February, 1905. Thirteen branch societies, or societies with similar aims and purposes, have since been formed in different cities and States, and others are now projected or are in process of formation.

*The Need of this Work.*—The facts which furnish the motive to this movement are:

1. *The Enormous Prevalence of These Diseases and Their Significance as a Danger to the Public Health.*—In point of prevalence they vastly overshadow all other infectious diseases, both acute and chronic combined. It is a conservative estimate that fully one-eighth of all human disease and suffering comes from this source. Moreover, the incidence of these diseases falls most heavily upon the young during the most active and productive period of life. It is a fact worthy of consideration that every year in this country

770,000 males reach the age of early maturity, that is, they approach the danger zone of initial debauch. It may be affirmed that under existing conditions at least 60 per cent, or over 450,000, of these young men will some time during life become infected with venereal disease, if the experience of the past is to be accepted as a criterion of the future. Twenty per cent of these infections will occur before their twenty-second year, 50 per cent before their twenty-fifth, and more than 80 per cent before they pass their thirtieth year. These 450,000 infections, be it understood, represent the venereal morbidity incident to the male product of a single year. Each succeeding group of males who pass the sixteenth year furnishes its quota of victims, so that the total morbidity from this constantly accumulative growth forms an immense aggregate.

2. *Dangers to the Innocent Members of Society.*—This was the chief impelling motive to the organization of this movement. Of the large proportion of men who contract venereal disease, many of them carry this infection into the family. Unfortunately, these diseases are markedly accentuated in virulence and danger to the wife and mother in fulfilling the functions for which marriage was instituted. There is abundant statistical evidence to show that 80 per cent of the deaths from inflammatory diseases peculiar to women, 75 per cent of all special surgical operations performed on women, and over 60 per cent of all the work done by specialists in diseases of women, are the result of gonococcus infection. In addition, 50 per cent or more of these infected women are rendered absolutely and irremediably sterile, and many are condemned to lifelong invalidism. Every year in this country thousands of pure young women are infected in the relation of marriage, their conceptional capacity destroyed, the aspirations which center in motherhood and children swept away, or the holy office of maternity desecrated by the bringing forth of tainted, diseased or dead children, and the women themselves often ruined in health or condemned to mutilation of their maternal organs to save their lives.

3. *Dangers to the Offspring.*—The effects of these diseases introduced into marriage are not measured alone by the danger to the life and health of the mother, but are still further manifest in their danger to the offspring. Fully 80 per cent of the ophthalmia which blots out the eyes of babies, and 20 to 25 per cent of all blindness, is caused by gonococcus infection. Syphilis is the only disease which is transmitted to the offspring in full virulence. Its effect upon the product of conception is simply murderous. Sixty to 80 per cent of all infected children die before being born or come into the world with the mark of death upon them. Those that finally survive—one in four or five—are the subjects of degenerative changes and organic defects which may be transmitted to the third generation.

Such are some of the undeniable and scientifically demonstrated dangers of a class of diseases, aptly designated as the "Great Black Plague," whose ravages have always been covered up and concealed from the public. There are some facts which it is more dangerous to conceal than to expose.

4. *Economic Significance.*—The fact that these diseases constitute the most potent factor in the causation of blindness, deaf-mutism, idiocy, insanity, paralysis, locomotor ataxia and other incapacitating and incurable affections, imposes an enormous charge upon the State and community. Millions of dollars are contributed to the support of defectives, but not a dollar for the dissemination of the saving knowledge which might prevent.

5. *Relation to Tuberculosis.*—It should be known that the spread of tuberculosis is not simply a question of seed and environment, but chiefly one of soil suitable for the development of the tubercle bacilli. It is a fact well recognized by the medical profession that syphilis, by lowering the vitality and weakening resistance, produces a condition favorable to the development of tuberculosis. Until the spread of syphilis is effectively checked the fight against tuberculosis will be but partially successful.

*Objects and Aims.*—The more immediate objects of the educational work undertaken by this society are: (1) The prevention of the large number of infections which occur in the young, the immature and the irresponsible through ignorance. (2) The preservation from infection of virtuous wives and innocent children who are powerless to protect themselves. (3) The prevention of the vast mass of disease and misery engendered in the descendants. It will thus be seen that this work, apart from its sanitary and economic importance, has a distinct humanitarian value.

*Remedial Measures.*—The basis of any intelligent scheme of prophylaxis is the adaptation of its measure to the causes of disease and the conditions under which it is spread. While there are other causes which contribute to the spread of these diseases, the basic cause is ignorance. The educational part of this society's program embraces as its most essential features:

1. The general dissemination of knowledge among the public, in proper and discreet manner, of the extent and dangers of these diseases, and their modes of contagion direct and indirect.

2. The enlightenment of the public respecting the social dangers of these diseases, especially to the innocent members of society through their introduction into marriage.

3. The education of young men in a knowledge of their physical selves and of the laws and hygiene of sex.

Since the ordinary channels of communication with the public and which serve for its enlightenment are not available, the only practical means of disseminating this knowledge is through lectures, conferences, pamphlets, printed slips and other educational literature.

Among other objects of this society's work is the correction of the causes of which these diseases are the outgrowth. Committees on "Treatment," "The Social Evil," and "Legislation," indicate the lines along which the society's work is conducted.

What measure of preventive value may be reasonably expected from this educational work? It is believed by medical men who are most competent to judge in these matters that the knowledge we seek to convey would be of inestimable benefit in preventing thousands of ignorant and reckless exposures to the dangers inseparable from immoral relations. Whatever may be the value of educational and moral training as a preservative against voluntary exposure to infection, it would certainly constitute a valuable prophylactic measure against the introduction of these infections into marriage. The vast majority of infections in married life are effected through ignorance—the opinion of all physicians is concurrent upon this point. The average man is not a criminal; he does not wreck the life and health of his wife and children knowingly and wilfully. In most cases he does it through ignorance of the nature and terrible consequences of his disease, ignorance of the prolonged duration of its contagious activity, and especially ignorance of the fact that it is often infectious after apparent cure.

It is also believed that publicity of these evils, especially the havoc wrought in the home and family, can not fail to create a public sentiment in favor of this work, which will lead to a sympathetic and active co-operation on the part of all men and women interested in the social welfare.

This society has been unable to utilize many opportunities for the enlargement and extension of its work in various directions from the lack of necessary funds. It is hoped that this difficulty will disappear with a more general knowledge among public-spirited citizens of the value and importance of this work.

PRINCE A. MORROW, M. D.,
66 West 40th Street.

